# Establishment of an interdisciplinary vascular anomalies program in Tanzania, East Africa

**DOI:** 10.3389/fmed.2022.1056539

**Published:** 2023-01-10

**Authors:** Daniel Puhr-Westerheide, Max Masthoff, Jay Shah, Alina Krechel, Mwivano Shemwetta, Azza A. Naif, Ofonime N. Ukweh, Ziad Abdul, Abizer Sarkar, Balowa Musa Baraka, Furaha Malecela, Praygod Justin Lekasio, Latifa Rajab, Abbas Mungia, William Sianga, Karim P. Manji, Eric M. Mbuguje, Sarah Khoncarly, Frank J. Minja, Fabian M. Laage Gaupp, Moritz Wildgruber

**Affiliations:** ^1^Department of Radiology, University Hospital, Ludwig Maximilian University of Munich, Munich, Germany; ^2^Clinic for Radiology, University Hospital Muenster, Münster, Germany; ^3^Division of Interventional Radiology and Image-Guided Medicine, Department of Radiology and Imaging Sciences, Emory University School of Medicine, Atlanta, GA, United States; ^4^Division of Pediatric Radiology, Department of Radiology and Imaging Sciences, Emory University School of Medicine, Atlanta, GA, United States; ^5^Department of Radiology, Muhimbili University of Health and Allied Sciences, Dar es Salaam, Tanzania; ^6^Department of Radiology, Muhimbili National Hospital, Dar es Salaam, Tanzania; ^7^Department of Dental Services, Oral and Maxillofacial Surgery, Muhimbili National Hospital, Dar es Salaam, Tanzania; ^8^Department of Neonatology, Muhimbili University of Health and Allied Sciences, Dar es Salaam, Tanzania; ^9^Department of Interventional Radiology, Michigan Medicine, University of Michigan, Ann Arbor, MI, United States; ^10^Department of Radiology and Imaging Sciences, Emory University School of Medicine, Atlanta, GA, United States; ^11^Section of Interventional Radiology, Department of Radiology and Biomedical Imaging, Yale New Haven Hospital, New Haven, CT, United States

**Keywords:** radiology, interventional/education, health services needs and demand, developing countries, Tanzania, interventional/pediatrics, vascular malformations

## Abstract

**Purpose:**

The aim of this project is the sustainable implementation of a vascular anomalies (VA) program in Tanzania.

**Materials and methods:**

In 2021 the first interdisciplinary VA program was initiated at Muhimbili National Hospital (MNH), Dar Es Salaam, Tanzania in a stepwise approach. During the planning phase the clinical need for minimally-invasive therapies of VAs and the preexisting structures were assessed by the local Interventional Radiology (IR) team at MNH. During the initiation phase, an IR team from two German VA centers joined the interdisciplinary team at MNH for clinical workup, image-guided procedures and follow-up. VA patients were recruited from existing patient records or seen at clinics as *de novo* presentations following nationwide advertisement. In the post-processing phase joined online conferences for follow-up and support in management of new patients were established. Further follow-up was supported by attending providers from other established VA centers, traveling to bolster the primary operators of MNH.

**Results:**

The first interdisciplinary VA program was successfully launched in Tanzania. Minimally-invasive treatments were successfully trained, by performing ultrasound-guided sclerotherapy with polidocanol and bleomycin in twelve patients with slow-flow malformations, one endovascular embolization of a high-flow malformation, and medical treatment of an aggressive infantile hemangioma. Regular online follow-up presentations have been initiated. Follow-up evaluation and required treatment was sustained when appropriate.

**Conclusion:**

The presented “hands-on” training set the ground for the first interdisciplinary VA program in Tanzania. This framework is expected to establish comprehensive and sustainable care of patients with VAs in East Africa and can serve as a blueprint for other sites.

## 1. Introduction

Interventional radiology (IR) has evolved rapidly in high-income countries, offering a broad spectrum of minimally-invasive diagnostic and therapeutic options. However, IR is not available to the majority of patients in low and middle-income countries (1). While already severe in adult IR, this disparity becomes even more evident in pediatric IR. Tanzania, an East African country with 60 million people, has a growth rate of approximately 3% and a birth rate of 4.9 births per woman and about 50% of the population being below the age of 18 years (2). Considering these demographics, vascular anomalies (VAs) can commonly be found in Tanzania. In general, the majority of vascular malformations are venous malformations (VM, ∼70%) with 1-2 in 10,000, followed by lymphatic malformations (LM, ∼12%), arterio-venous malformations (AVM, ∼8%) and others, such as capillary or mixed types (3, 4). Overall, 16-48% of VAs can be found in the head or neck region (5). Incorrect classification of VAs as hemangiomas and ineffective treatment is common even in high-income countries, including the risk of harmful treatments of VA (6, 7). In Tanzania, treatment options of VA until now were limited to propranolol, surgery, or interstitial bleomycin injection. Traditional treatments of VAs can be found in rural areas of Tanzania as reported by VA patients, in some cases including incision of the skin and subcutaneous tissues, which puts patients at high risk for infection or bleeding as well as disfiguring scarring. Interdisciplinary minimally-invasive IR treatment strategies of these malformations have not been established in Tanzania and neighboring countries until now (4, 8–11). Therefore, after the successful implementation of the first IR service and training program in Tanzania in October 2018 (12), a multi-departmental training camp to initiate an interdisciplinary VA program in East Africa at MNH was undertaken in November 2021. MNH is a national referral hospital in Daressalam, Tanzania, with a capacity of 1600 beds including surgical, internal medicine, pediatric, neurology and neurosurgery as well as radiology and interventional radiology service.

The goal of this program is to establish an interdisciplinary framework for the diagnosis and treatment of VAs on site, to train the IR team at MNH in interventional therapies of the common types of VA, and to create an interdisciplinary platform for follow up and future therapies.

## 2. Materials and methods

### 2.1. Planning phase

In preparation for the on-site training, monthly virtual meetings with the interventional radiology trainees and graduates at MNH, the program coordinator, former visiting IR physicians, and the visiting pediatric IR team were started six months prior.

Videoconferences focused on the following topics:

1. Education in VA diagnostics, treatment (medical, interventional, surgical) and follow-up with lectures and journal club.
2. Recruitment of VA patients from the entire nation – phone calls with regional and district hospitals in Tanzania, review of VA patient records from OMFS.
3. Establishment of a structured VA reporting form for initial patient presentations, treatment documentation and follow-up (Supplementary material).
4. Organizing materials for VA treatment (sclerosants, liquid embolics, catheters) due to very limited access to IR materials in Tanzania.
5. Case discussions for treatment planning during the visit. For case discussions patients were completely anonymized.

For the first implementation of the program, the focus was set to the treatment of facial vascular anomalies. Therefore, the local interdisciplinary VA team was established as followed: (1) interventional radiologists performing pre-interventional clinical assessments and sonography in clinics and the image-guided sclerotherapy or embolization, respectively, (2) oral and maxillofacial surgeons for patient recruitment, clinical assessment, support during image guided therapy, pre- and post-interventional care of patients on the ward and surgical treatment in case of necrosis or other complications, (3) pediatricians and pediatric surgeons for patient recruitment, pre- and post-interventional care of patients on the ward, management of potential complications or second-step resection after complete occlusion of the malformation, and (4) neonatologists for medicamentous and conservative treatment of patients with congenital vascular anomalies, especially hemangiomas.

**FIGURE 1:**
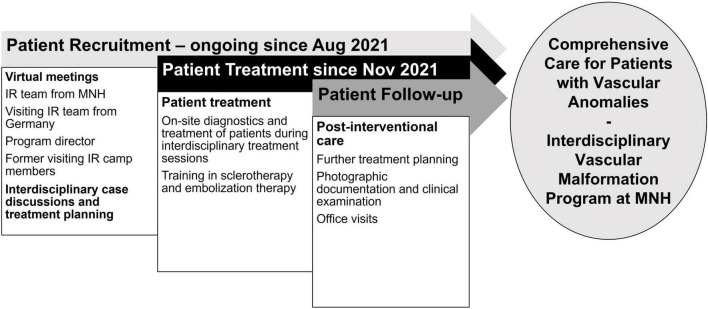
Timeline of the establishment of a vascular anomalies treatment program in Tanzania: preparation for the two-week camp, goals during hands-on training, and post-interventional patient care. IR = Interventional Radiology; MNH = Muhimbili National Hospital.

The visiting IR team consisted of three interventional radiologists and one IR technologist from two academic VA centers in Germany. Recruitment of VA patients for this project was initiated three months prior to the teaching visit in August 2021 in close coordination with the collaborating departments (Figure 1).

### 2.2. IR devices

IR devices are expensive and up to date there is no dedicated market for IR material in Tanzania. Therefore, this program largely depends on devices brought along by the visiting teams which are product donations from cooperating companies in the respective home countries. For the future, lower price devices and cooperation with companies are crucial to establish an official selling of material to Tanzania for long term sustainability.

### 2.3. On-site training phase

In November 2021 the visiting IR team joined the local IR team at MNH. Several lectures including grand rounds on diagnosis and treatment of VA were conducted to educate and integrate IR in the local interdisciplinary setting for this topic. Patients recruited during the planning phase were seen in interdisciplinary clinic, which included the local IR team and collaborating departments. Thorough patient history, clinical examination, review of cross-sectional imaging, and ultrasound assessment of vascular anomalies were performed under supervision of the visiting team with a focus on differentiating characteristics of slow-flow versus high-flow vascular malformations. Imaging protocols and characteristics of VAs on CT and MRI were discussed and trained with the local IR team and radiographers. For diagnosis in accordance with ISSVA classification, consensus was reached between two visiting IR attendings experienced in VA diagnosis and treatment and two local trainees and/or attending IR physicians. Individual cases were discussed by the local IR team, collaborating disciplines (e.g., oral and maxillofacial surgery, OMFS), and the visiting IR team for specific treatment planning. Based on the final recommendations of the interdisciplinary VA clinic, interventions were performed by the local IR team in collaboration with the various surgical disciplines under close supervision of the visiting IR team. Informed consent was obtained from the patient or parent before undergoing treatment.

### 2.4. Technical implementation

VMs were treated primarily with an ultrasound-guided approach (due to limited availability of fluoroscopy). In case of VMs, ultrasound needles were placed intravascularly within the dysplastic venous network under general anesthesia and a sclerosant was injected under continuous ultrasound visualization. All sclerotherapies were performed by local IR team members (trainees and faculty members) under supervision of the visiting IR team. Embolization of fast-flow malformations was performed in a dedicated angiosuite, equipped with an angiography unit (Artis Zee, Siemens Healthineers, Forchheim, Germany). Embolization of a high flow arteriovenous malformation (AVM) was trained using liquid embolic material.

### 2.5. Post-treatment phase

All patients were admitted to the hospital and observed for at least one day after the procedure to guarantee sufficient pain management and to control swelling in the head and neck region as the infrastructure for specialized outpatient care is not available in Tanzania. Training included interdisciplinary ward rounds for follow-up management of VA patients. An early follow up was performed after 4 weeks in clinic or remotely via telephone in cases where patients came from far away. Further, management of patients for follow-up appointments and/or additional procedures such as repeated sclerotherapy or surgery after embolization was planned. After completion of the onsite training with the visiting IR team, sustainability was ensured by regular conferences of the MNH local and visiting IR team regarding patients’ follow-up or, if needed, support in management of new patients.

### 2.6. Follow-up and retreatment phase

All patients treated during initial implementation were in regular contact with a VA team member (OMFS team or IR team). Follow-up of patients at clinics at MNH was planned 4 weeks after the initial therapy including clinical examination and ultrasound assessment. If patients came from far away also telemedicine-type follow up (video calls) was used. Follow-up evaluation was done in conjunction with the multidisciplinary team, and primarily managed by MNH local IR team centering on physical exam (development of the size of the malformation, areas of post-interventional necrosis?) and ultrasound (residual perfused parts of the malformation, low flow vs. high flow). In addition to these findings, foundational outcomes such as pain, cosmesis and function were central to each evaluation and need for follow up. Follow-up presentation was also used to assess the need for further treatment sessions. The majority of these patients for which follow-up treatment was required were recalled, at the time when the next VA trained IR (this time from the USA) was based at MNH. For patients requiring follow-up treatments, the treatment plan was determined, and the patient was either admitted or referred for return during dedicated vascular anomaly operating theater block time. Similar to established VA programs in high-income countries, sclerotherapy is best prescribed as serial therapy in order to gain maximum results and minimize complications. Planned follow-up may require a different sclerosant or method for safety, or in the case of LMIC settings - availability of sclerosants. Continuity of care and management was primarily performed by MNH IR team members with consultation by the visiting IR team. The follow-up plan for each patient was determined prior to discharge from the post-operative ward. Imaging and gross pictures are kept and managed by MNH the local IR team as the primary operator.

## 3. Results

The first interdisciplinary VA program in East Africa was successfully launched, including establishment of protocols guiding patient referral, evaluation, state of the art treatment, and follow-up. Pre-treatment case discussions and patient recruitment was key for the success of the interdisciplinary VA camp. During the first days of the VA camp patients were seen in clinics and cross-sectional imaging was reviewed. In parallel, the treatments started for low flow malformations.

**FIGURE 2:**
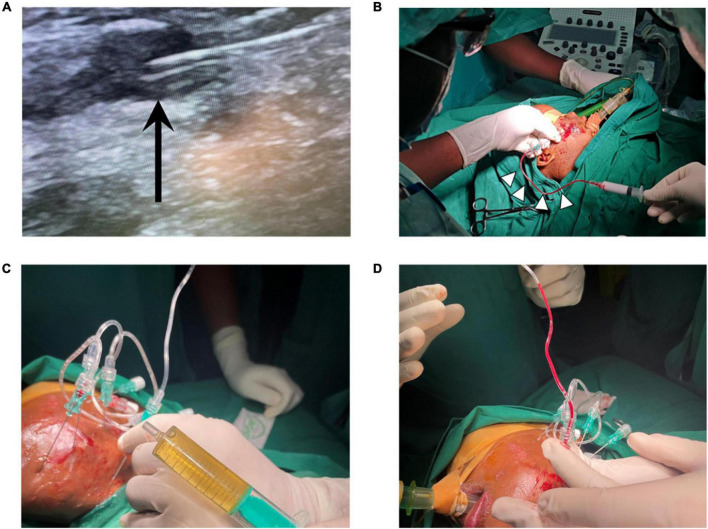
**(A)** Ultrasound-guided needle placement in dysplastic veins of a VM, black arrow points at needle tip. **(B)** Aspiration of blood (white arrow heads) confirms needle position within dysplastic VM vessels prior to sclerotherapy with polidocanol foam. **(C)** Aspiration of lymph fluid and **(D)** aspiration of blood in a mixed-type lymphatic and venous malformation, treatment with bleomycin (for lymphatic cysts) and polidocanol (for venous components). VM = venous malformation.

During this initial training visit a total of eight children and five adults with VAs were treated with an image-guided, minimally invasive approach at MNH. All malformations treated during this first VA camp were localized in the head, face, or neck. Eleven patients presented with VMs (84.6%), two with venous-lymphatic malformations (15.4%), and one with a large high flow AVM of the forehead. The different nature of these vascular anomalies contributed significantly to the learning experience as different treatment approaches were trained (e.g., draining of LMs with subsequent percutaneous image-guided bleomycin sclerotherapy, endovascular embolotherapy for AVM). These cases illustrated well the aim and benefits of image-guided sclerotherapy. All 13 patients received the first session of intravascular percutaneous, image-guided sclerotherapy during this initial teaching visit, with a plan for subsequent sessions during future VA camps with supporting visiting teams. Teaching points such as the correct needle placement and distribution of the sclerosing agent was imaged in real-time on ultrasound Figures 2A, B). The key teaching points were safe intravascular ultrasound-guided needle placement within the dysplastic vessels, aspiration of blood/lymphatic fluid to ensure stable intravascular position of the needle, and subsequent injection of sclerosant with real-time monitoring under ultrasound. Two patients had malformations with lymphatic and venous components (LVM). The lymphatic cysts were drained and bleomycin (up to 10 mg in a concentration of 1 mg/ml) was injected into the cavities (Figure 2C). In these cases, the patient’s charts were reviewed carefully before therapy to ensure that the bleomycin lifetime dose was not violated. The administered bleomycin dose was meticulously documented to assure correct dose calculations within the allowed lifetime dose during follow up treatments. These teaching points aimed for sustainability and safe follow up treatments for the patients. One patient (age: 10 months) presented with a high-flow arteriovenous malformation on the forehead with growth progression. In this case, successful embolization with liquid embolics was performed under general anesthesia followed by surgical resection in March 2022. Teaching points were safe arterial access (4F) through the femoral artery, cautious navigation into the supraaortic arteries avoiding any risks of air embolism, superselective positioning of a microcatheter within the maxillary artery, and embolization of the nidus via the arterial feeders of the AVM in plug-and-push technique with a liquid embolic agent, while avoiding non-target embolization.

**FIGURE 3:**
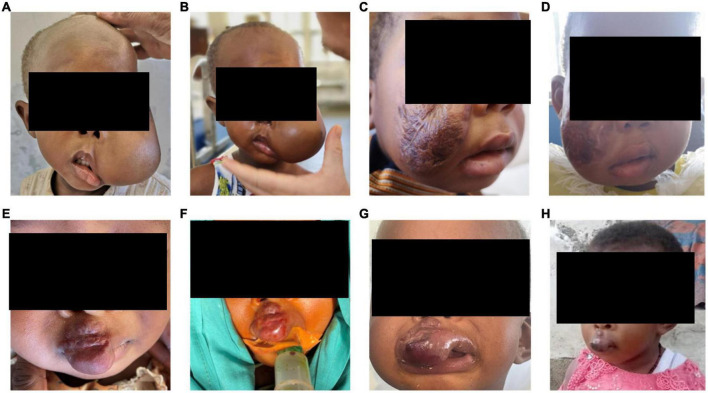
**(A)** Pre-procedural images of a 2-year-old girl who presented to the VA clinic with left cheek swelling due to a VM of the left cheek and face with involvement of the lateral border of the left eye. **(B)** Post-interventional swelling occurred in this patient as expected. **(C)** Pre-procedural images of a 3-year-old girl with a VM of the right cheek, the patient underwent prior traditional medicine treatment leaving extensive scars on the right cheek. **(D)** Post-interventional swelling occurred as expected in this patient. **(E,F)** Pre- and intra-procedural images of a 2-year-old girl with a VM on the upper lip extending up to the right nostril. **(G)** After the procedure a swelling occurred, and a slight dark discoloration demarcated on the lip. **(H)** The small necrosis healed quickly and the proportion below the right nostril already shrunk significantly. All patients need further treatment sessions and close follow-up. VA = vascular anomalies.

**FIGURE 4:**
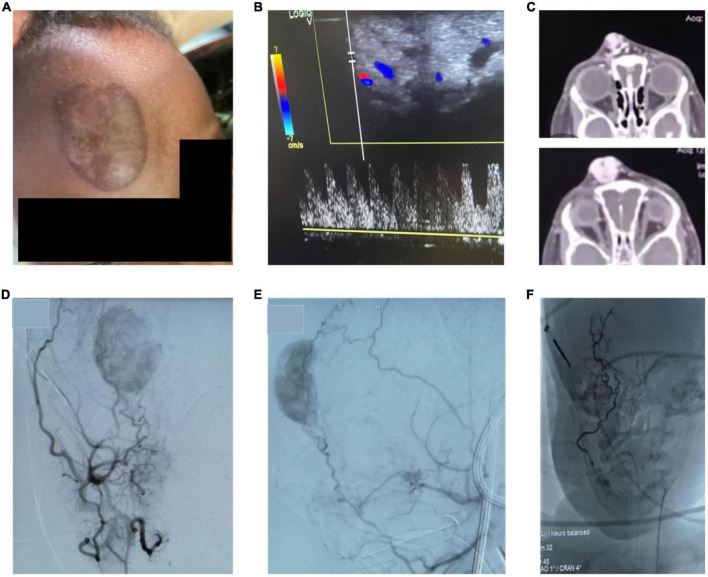
**(A,B)** Pre-procedural picture of a clinical examination showing a pulsatile, warm AVM of the forehead with corresponding high flow on doppler ultrasound. **(C)** CT-images with contrast show a highly vascularized lesion with arterial feeders and venous drainage via the ophthalmic veins. **(D,E)** Angiographic images of the AVM by contrast administration through the external carotid artery confirm the facial artery as the major feeding vessel. Further, the frontal branch of the temporal artery has small feeding branches. **(F)** After embolization of the main feeding vessels with liquid embolic material occluding the arterial vascular network, the intravascular cast formed by the liquid embolic agent can be appreciated. The patient underwent subsequent surgical resection of the lesion.

Patients were admitted to the hospital for minimally-invasive treatment and in-patient treatment was provided for 2-4 days after the interventional therapy, depending on the localization and nature of the vascular malformation. In-patient follow up included laboratory and clinical examinations. No infectious complications and no fever occurred in patients treated with percutaneous sclerotherapy. On the day after sclerotherapy, post-interventional swelling was observed in all patients as expected (pre- and post-treatment examples shown in Figure 3). One patient underwent sclerotherapy of a venous malformation on the upper lip and developed a small, localized necrosis, which did not require further treatment other than close follow up and careful wound management (Figures 3E–H). Another patient developed more severe necrosis of the cheek and nose tip with the need of wound management and plastic surgery. This complication likely could have been avoided with fluoroscopy control (which was not available for sclerotherapy at that time at MNH). However, this offered an opportunity to train management of typical complications with the local interdisciplinary VA team. Access to fluoroscopy for the MNH IR team has since improved and will continue to expand in coming years. No embolization-related complications were observed after the high flow AVM embolization on the forehead. Pre-therapeutic images and angiography are shown in Figure 4. However, the child developed a pneumonia in the first week after the therapy which was most likely attributed to the difficult intubation and prolonged mask ventilation in preparation of the procedure. The child had to be treated on ICU for 3 days, had a quick recovery and was discharged after 4 days. No other complications such as reflex syncope occurred in patients treated with percutaneous sclerotherapy or endovascular embolization, respectively. Details on observed adverse events can be found in Table 1.

**TABLE 1:** Patient characteristics and procedural details.

ID	Age [y]	Sex	Diagnosis	Localization	Previous therapy	Kind of previous therapy	Treatment	Sclerosing agent	Complications	Plt (x10^3^/μ L) WBC (x10^3^/μ L)	Days in hospital	Follow up in months
1	1	F	VM	Upper lip	Yes	Unguided bleomycine injection	PS	Polidocanol (2 ml)	Small localized necrosis	467/7.4	2	10
2	2	F	VM	Left cheek	Yes	Unguided bleomycine injection	PS	Polidocanol (6 ml)	None	273/3.6	2	10
3	2	F	VM	Right cheek	Yes	Unguided bleomycine injection and traditional medicine	PS	Polidocanol (4 ml)	None	244/8.3	2	10
4	8	F	VLM	Mandible/chin	Yes	Unguided bleomycine injection	PS	Polidocanol (4 ml) Bleomycine (28 mg)	None	306/6.2	2	10
5	4	M	VM	Left cheek	Yes	Unguided bleomycine injection	PS	Bleomycin (10 mg)	None	116/10.3	2	10
6	5	F	Tufted angioma/VM	Left cheek	Yes	Unguided bleomycine injection	PS	Polidocanol (4 ml)	None	382/7.4	2	10
7	52	M	VM	Right cheek	No	-	PS	Polidocanol (8 ml)	Necrosis	262/8.2	2	10
8	20	M	VM	Left cheek	Yes	Unguided bleomycine injection	PS	Polidocanol (1.7 ml)	None	241/4.3	2	10
9	15	F	VM	Right cheek	No	-	PS	Polidocanol (8 ml)	None	354/5.1	3	10
10	64	F	VLM	Right cheek and lip	No	-	PS	Polidocanol (0.7 ml)	None	171/6.5	2	10
11	32	M	VM	Right cheek	Yes	Unguided bleomycine injection	PS	Polidocanol (6 ml)	None	104/3.4	2	10
12	30	F	VM	Chin and both cheeks	Yes	Unguided bleomycine injection	PS	Polidocanol (6 ml)	None	253/3.2	2	10
13	0 (11 mo)	F	AVM	Forehead	No	-	Embolization	EVOH (1.2 ml)	Pneumonia	67/6.8	4	10

F: female; M: male; mo: months; y: years; VM: venous malformation; AVM: arteriovenous malformation; PS: percutaneous sclerotherapy; EVOH: ethylene vinyl alcohol; Plt: platelet count; WBC: white blood cell count.

**FIGURE 5:**
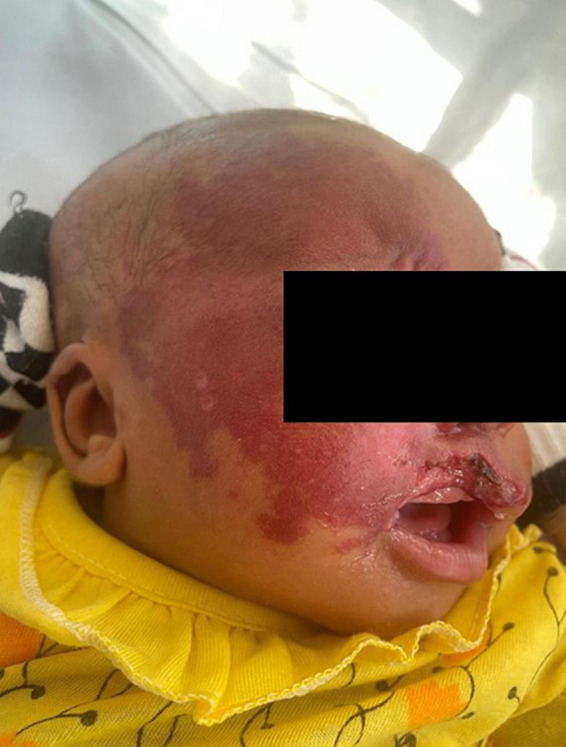
Neonate with an aggressive infantile hemangioma of the cheek and periorbital region leading to a localized steal-effect with subsequent necrosis on the upper lip. Conservative treatment with propranolol p.o. (hemangiol starting dose 0.5 ml for 3 kg child) was initiated and further active surveillance was chosen as treatment strategy for this patient.

One patient on the neonatology ward presented with an aggressive infantile hemangioma of the cheek and periorbital region leading to a localized steal-effect with subsequent necrosis on the upper lip (Figure 5). Conservative treatment with propranolol p.o. (hemangiol starting dose 0.5 ml for 3 kg child) was initiated and further active surveillance was chosen as treatment strategy for this patient.

Of the initial 13 patients treated, 12 (92.3%) patients reported improvement of symptoms and only one (7.7%) showed no change in symptoms after therapy at 10 months follow-up. Long-term sustainability of the VA program in Tanzania has been secured by addressing several points: First, treated patients were followed up by the local IR team. Second, ongoing close communication between the visiting IR team, other pediatric IR worldwide and the local IR team via digital (video-) platforms to discuss further treatments and presentation of new patients was ensured. Third and most importantly, on site visits of further specialized pediatric IR teams every two to three months were organized. Until today (10/2022) three further pediatric IR teams have continued to build the VA program at Tanzania onsite. Within the first 12 months after the visit of the first pediatric IR team, a total of 36 further VA procedures were performed at MNH. Fourth, a cooperation with the Society of Pediatric Interventional Radiology (SPIR) was established including reduced membership fees and annual meeting discount for conference participation to MNH IR trainees and graduates.

## 4. Discussion

Considering the increasing demand for IR procedures in low and middle income countries, strategies to provide structured, accredited, and sustainable on-site training programs aiming to supply fully trained interventional radiologists in these settings are needed (1, 12, 13). While few such programs have been initiated over the past several years, demonstrating on a small scale that this can be achieved, there is need for massive upscaling of these efforts if IR care is to be provided worldwide (12, 14, 15). This especially holds true for pediatric IR, which struggles with even more challenges than adult IR, given the need for more specialized training and equipment to meet the needs of a young patient population (16). Thus, especially low- and middle-income countries and their overall younger populations are confronted with a near-total lack of cost-effective minimally-invasive care, resulting in major long-term consequences for individual patients and their societies at large. In this context, vascular anomalies, as common congenital disease affecting mostly children and adolescents, which can be treated by a range of IR procedures, provide a suitable starting point for implementation of pediatric interventional care along with a variety of other important pediatric IR treatments (e.g., biopsies, nephrostomy tube placement, abscess drainage). The presented study reports on such a program, establishing the first VA treatment center in Tanzania. Emphasis was placed on training the full cycle of care from clinical presentation to diagnosis, treatment, and follow-up. The most common types of VAs were diagnosed and treated by local IR team members during this half-year (and ongoing) theoretical and case-based educational program including a two-week VA training camp under the supervision of an experienced visiting IR team. The interdisciplinary framework for VA treatment was successfully launched for sustainable integrated VA treatment in close collaboration with OMFS, pediatric surgery, pediatrics and neonatology, thereby enabling Tanzanian IR team members to independently build up specialized VA-care over the total cycle of care. To ensure homogeneity of the patient cohort for teaching purposes, the focus of this first implementation was set to the treatment of facial VA, since most VA are located there (7). However, a majority of vascular malformations is also located at the extremities causing pain, swelling and immobility emphasizing the need to expand the vascular anomalies team in the future (7). In detail, the interdisciplinarity of the program will be further extended with the aim to include plastic and reconstructive surgery, ENT/head and neck surgery, orthopedics, dermatology and medical oncology as the next step. Exemplarily, first patients with VAs of the extremities have been treated at MNH in 2022. Thereby, the presented strategy on the one hand increases sustainability and independence from visiting IR specialists and on the other hand aims to foster IR not only as a diagnostic but also treating specialty in the center of interdisciplinary teams improving patient care for this complex condition. IR is recognized for providing minimally-invasive, cost-effective care (17). This holds especially true for VA treatment and investment in IR personnel and equipment may help to reduce costs in the long run in low- and middle-income countries such as Tanzania, as current strategies include sending VA patients to India for treatment on government expenses with costs of up to 10,000 USD per treatment. Moreover, it should be emphasized that having strong IR in one country will have positive effects on IR everywhere, but particularly in neighboring countries (13). For instance, the local IR team in Tanzania also consists of IR team members from other African countries such as Nigeria and Rwanda, established or plan to establish IR in their home countries after finishing the training program in Tanzania, making the program a nucleus for spreading IR in Africa (18). Maintenance of digital communication of experienced IR teams from the US and Europe with the local IR team members ensures ongoing education and consultation for new patients. Inclusion and active involvement of African IR team members in international societies such as the Society of Pediatric Interventional Radiology (SPIR) ensures access to scientific and educational content with the goal of keeping VA treatment algorithms up to date.

One major limiting factor for sustainability is the lack of a dedicated market for IR devices for Tanzania. Due to this fact, the IR program largely depends on devices brought along by the visiting teams which are product donations from cooperating companies in the respective home countries. For the future, lower price devices and cooperation with companies are crucial to establish an official selling of material to Tanzania for long term sustainability.

In summary, we report on the successful establishment of a sustainable, highly specialized IR guided interdisciplinary VA team in Tanzania, which can serve as a model for further expansion of pediatric and adult IR care in low- and middle-income countries. Establishment of minimally-invasive image-guided treatments in the field of congenital anomalies contributes to the major goal of providing equal care worldwide.

## Data availability statement

The original contributions presented in this study are included in the article/Supplementary material, further inquiries can be directed to the corresponding author.

## Author contributions

All authors contributed significantly to establish the VA program, planning the VA camp, therapy of patients, and writing of the manuscript.

## References

[1] Mollura D Soroosh G Culp M Averill S Axelrod D Baheti A 2016 RAD-AID conference on internastional radiology for developing countries: gaps, growth, and United Nations sustainable development goals. *J Am Coll Radiol.* (2017) 14:841–7. 10.1016/j.jacr.2017.01.049 28372963

[2] Worldometer. *Tanzania demographics 2020 (population, age, sex, trends).* (2022). Available online at: https://www.worldometers.info/demographics/tanzania-demographics/#urb (accessed on March 13, 2022).

[3] Behravesh S Yakes W Gupta N Naidu S Chong B Khademhosseini A Venous malformations: clinical diagnosis and treatment. *Cardiovasc Diagn Ther.* (2016) 6:557–69. 10.21037/cdt.2016.11.10 28123976 PMC5220204

[4] Sadick M Müller-Wille R Wildgruber M Wohlgemuth W. Vascular anomalies (Part I): classification and diagnostics of vascular anomalies. *Fortschr Röntgenstr.* (2018) 190:825–35. 10.1055/a-0620-8925 29874693

[5] Kobayashi K Nakao K Kishishita S Tamaruya N Monobe H Saito K Vascular malformations of the head and neck. *Auris Nasus Larynx.* (2013) 40:89–92. 10.1016/j.anl.2012.02.002 22534179

[6] Hassanein A Mulliken J Fishman S Greene A. Evaluation of terminology for vascular anomalies in current literature. *Plastic Reconstr Surg.* (2011) 127:347–51. 10.1097/PRS.0b013e3181f95b83 21200229

[7] Greene A Liu A Mulliken J Chalache K Fishman S. Vascular anomalies in 5621 patients: guidelines for referral. *J Pediatr Surg.* (2011) 46:1784–9. 10.1016/j.jpedsurg.2011.05.006 21929990

[8] Müller-Wille R Wildgruber M Sadick M Wohlgemuth W. Vascular anomalies (Part II): interventional therapy of peripheral vascular malformations. *Fortschr Röntgenstr.* (2018) 190:927–37. 10.1055/s-0044-101266 29415296

[9] Legiehn G Heran M. Classification, diagnosis, and interventional radiologic management of vascular malformations. *Orthopedic Clin North Am.* (2006) 37:435–74. 10.1016/j.ocl.2006.04.005 16846771

[10] Legiehn G Heran M. A step-by-step practical approach to imaging diagnosis and interventional radiologic therapy in vascular malformations. *Semin intervent Radiol.* (2010) 27:209–31. 10.1055/s-0030-1253521 21629410 PMC3036512

[11] Gibson C Barnacle A. Vascular anomalies: special considerations in children. *CVIR Endovasc.* (2020) 3:60. 10.1186/s42155-020-00153-y 32886264 PMC7474047

[12] Laage Gaupp F Solomon N Rukundo I Naif A Mbuguje E Gonchigar A Tanzania IR initiative: training the first generation of interventional radiologists. *J Vasc Intervent Radiol.* (2019) 30:2036–40. 10.1016/j.jvir.2019.08.002 31668662

[13] Kaufman J Sacks D Stainken B. Denied in Canada: why we need a global strategic plan for interventional radiology. *J Vasc Intervent Radiol.* (2008) 19:13–4. 10.1016/j.jvir.2007.11.001 18192462

[14] Kesselman A Gaupp F. RAD-AID global curriculum for interventional radiology. *Endovasc Today.* (2018) 17.

[15] Kline A Dixon R Brown M Culp M. Interventional radiology readiness assessment tool for global health. *JGR.* (2017) 3:2. 10.7191/jgr.2017.1035

[16] Shah S Binkovitz L Ho M Trout A Adler B Andronikou S. Pediatric radiology mission work: opportunities, challenges and outcomes. *Pediatr Radiol.* (2018) 48:1698–708. 10.1007/s00247-018-4221-x 30116834

[17] Masthoff M Schneider K Schindler P Heindel W Köhler M Schlüchtermann J Value improvement by assessing IR care via time-driven activity-based costing. *J Vasc Intervent Radiol.* (2021) 32:262–9. 10.1016/j.jvir.2020.09.017 33139185

[18] Novasys.io E ku. Rwanda conducts first vascular interventional radiology procedure at king faisal hospital. Kigali: King Faisal Hospital. (2022).

