# Supplementary figures and images for: Establishment of an interdisciplinary vascular anomalies program in Tanzania, East Africa
Source: Front Med (Lausanne). 2023 Jan 10;9:1056539. doi: 10.3389/fmed.2022.1056539 (PMC9871613; doi:10.3389/fmed.2022.1056539)

Supplemental Material


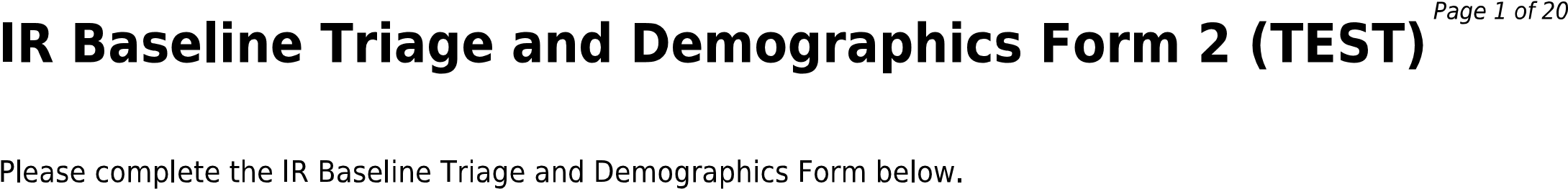


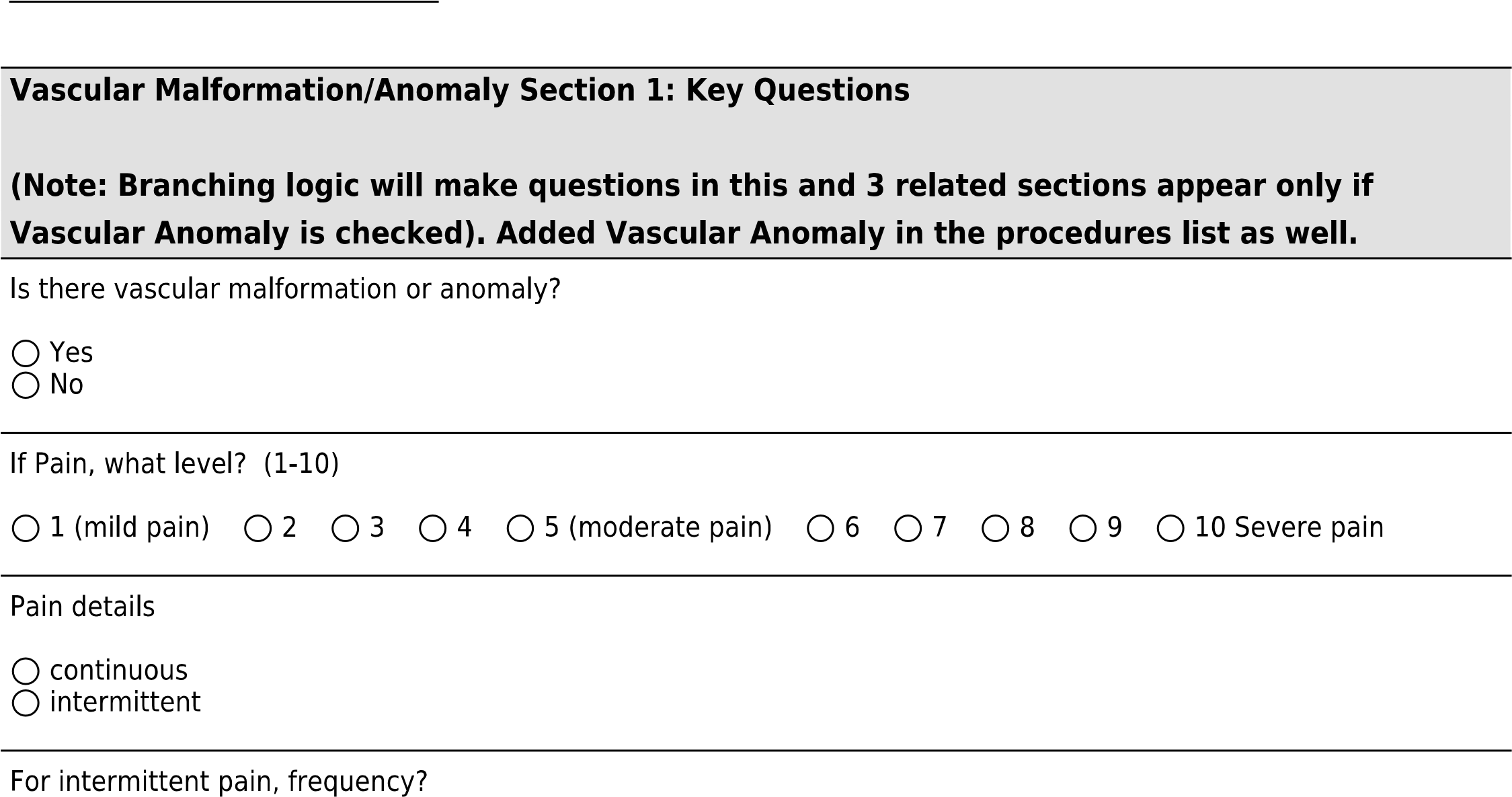


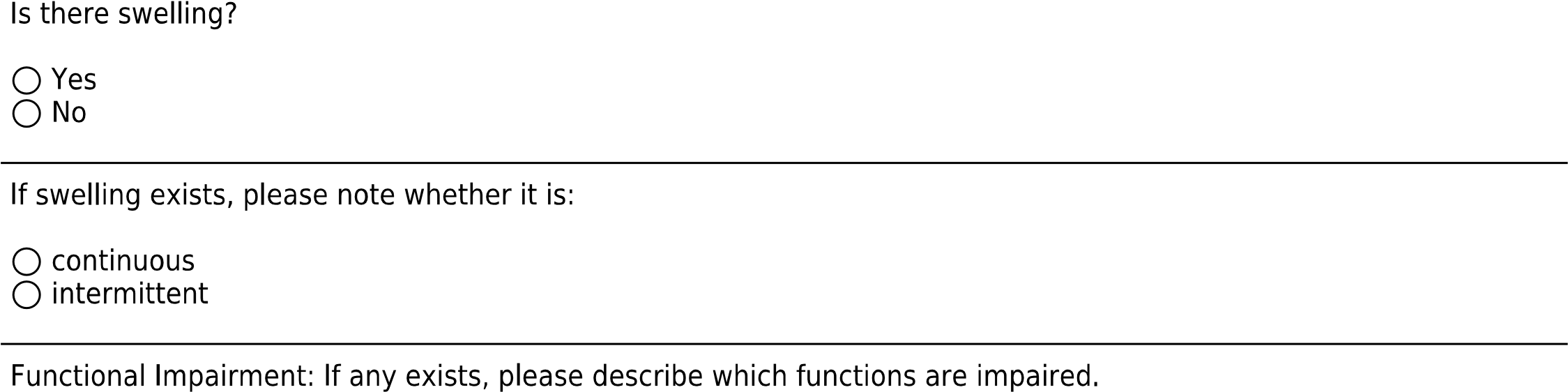


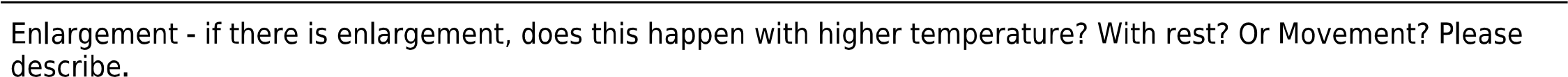


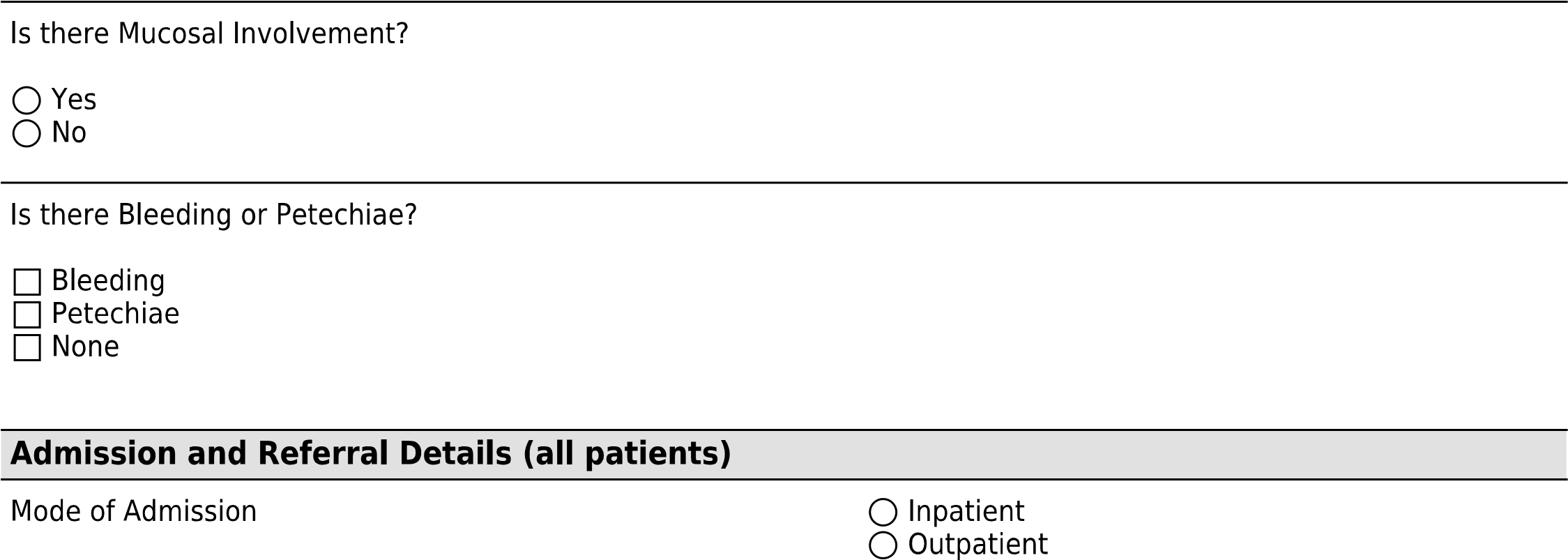


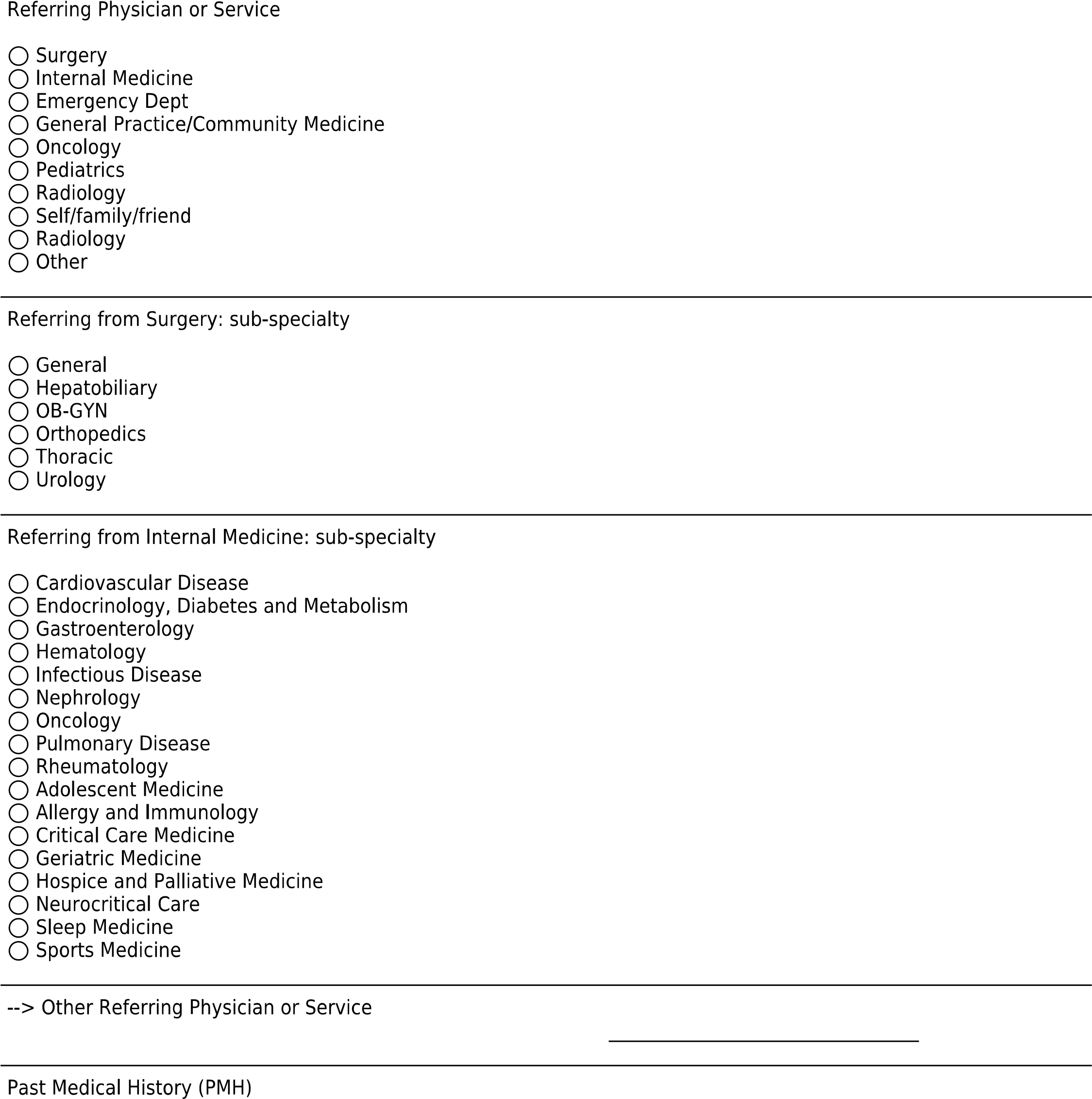


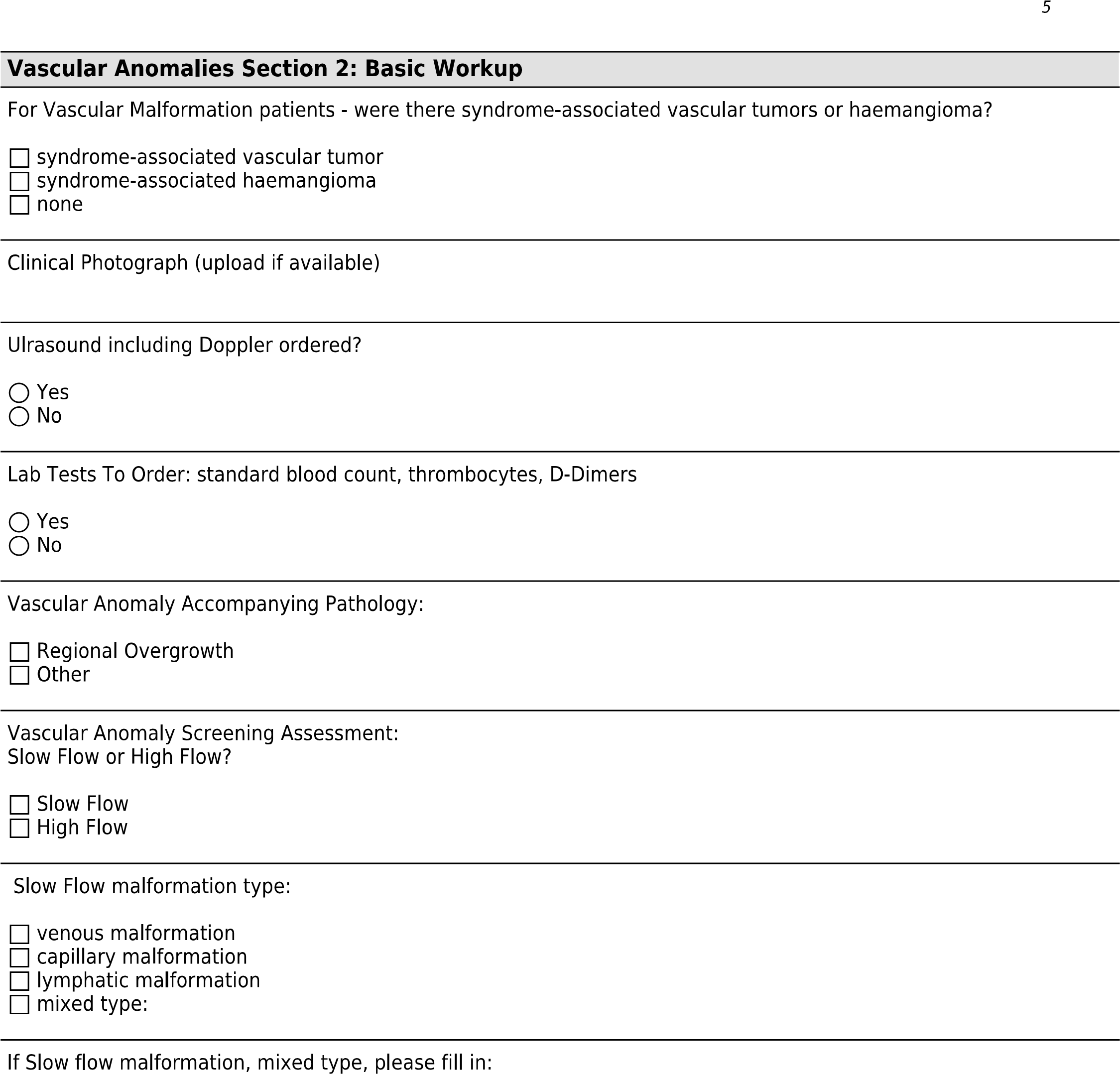


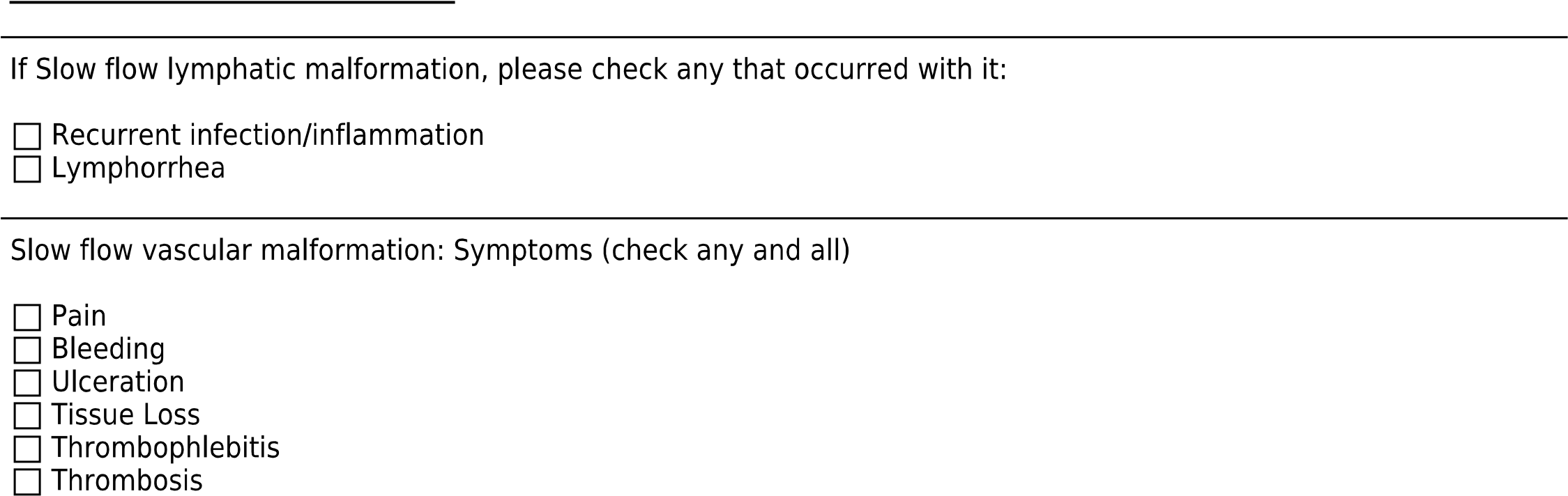


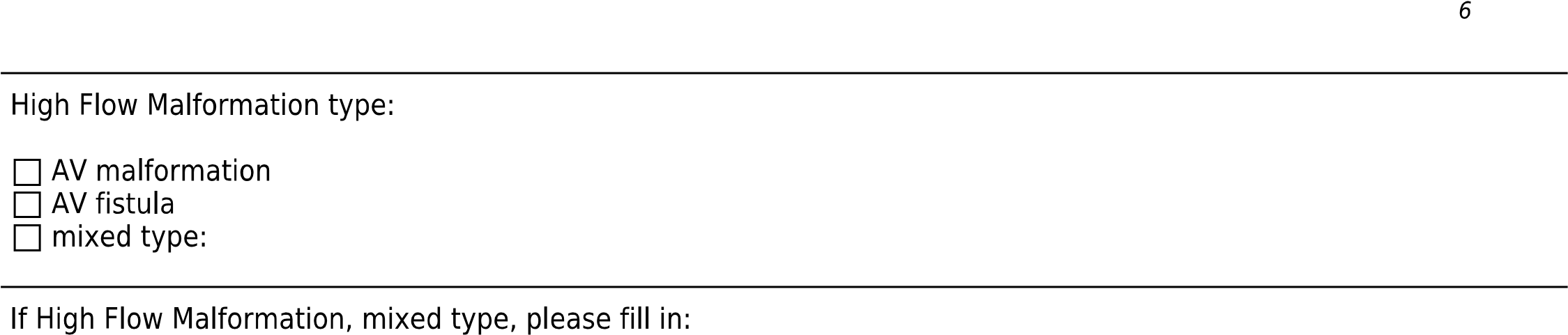


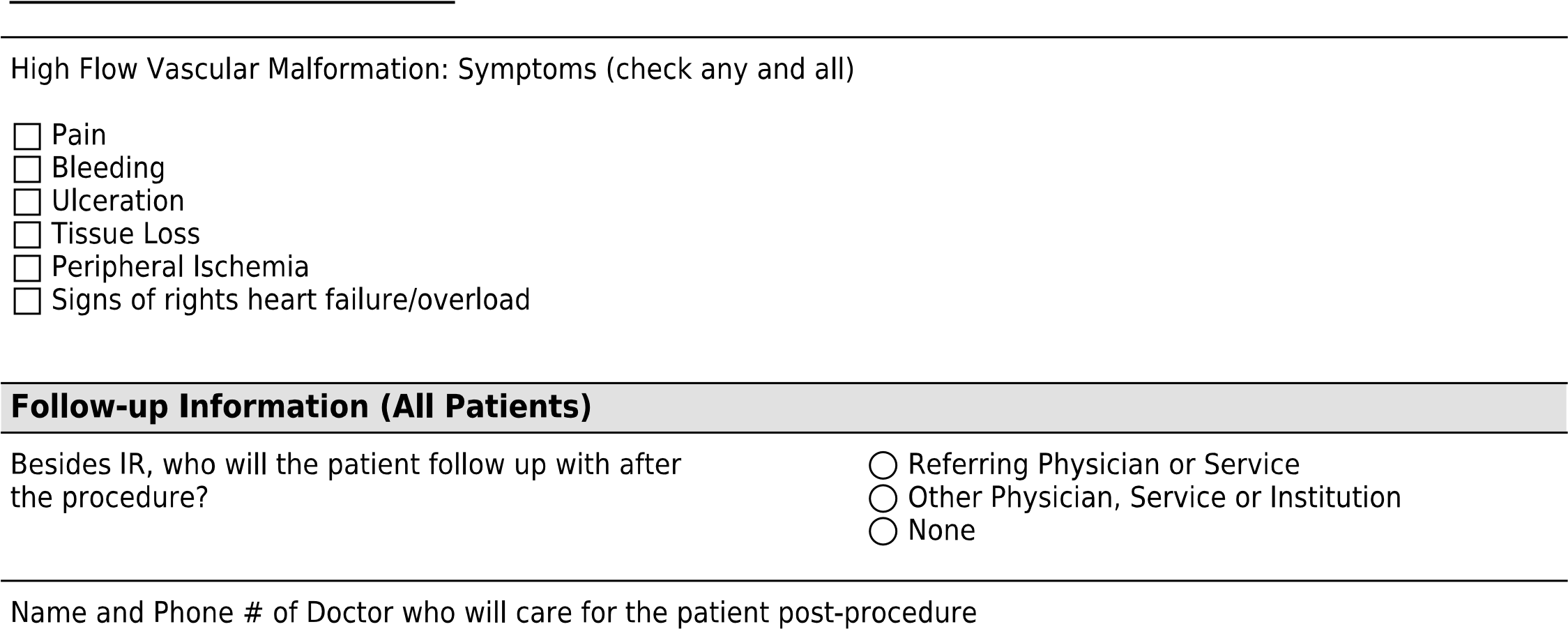


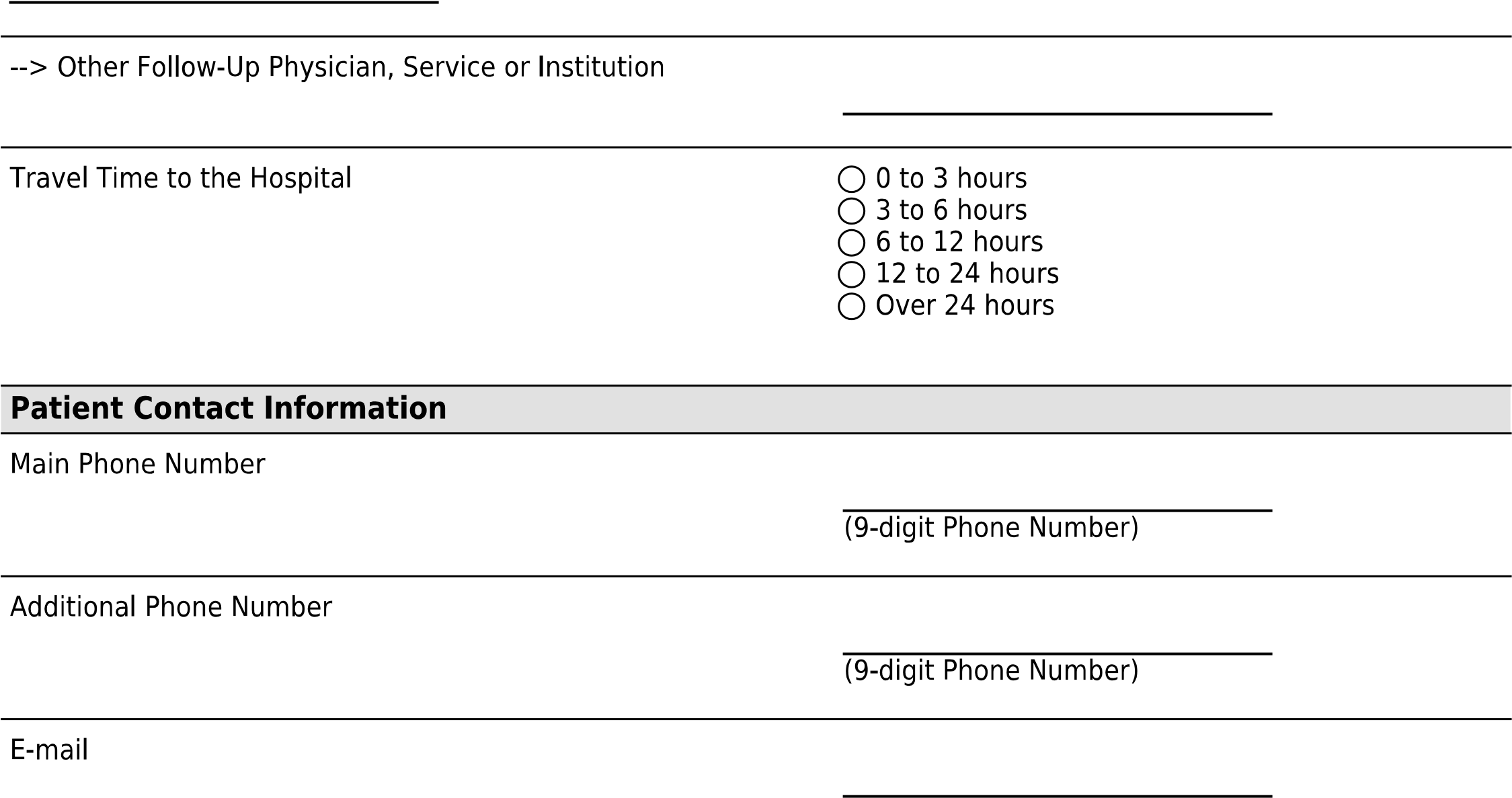


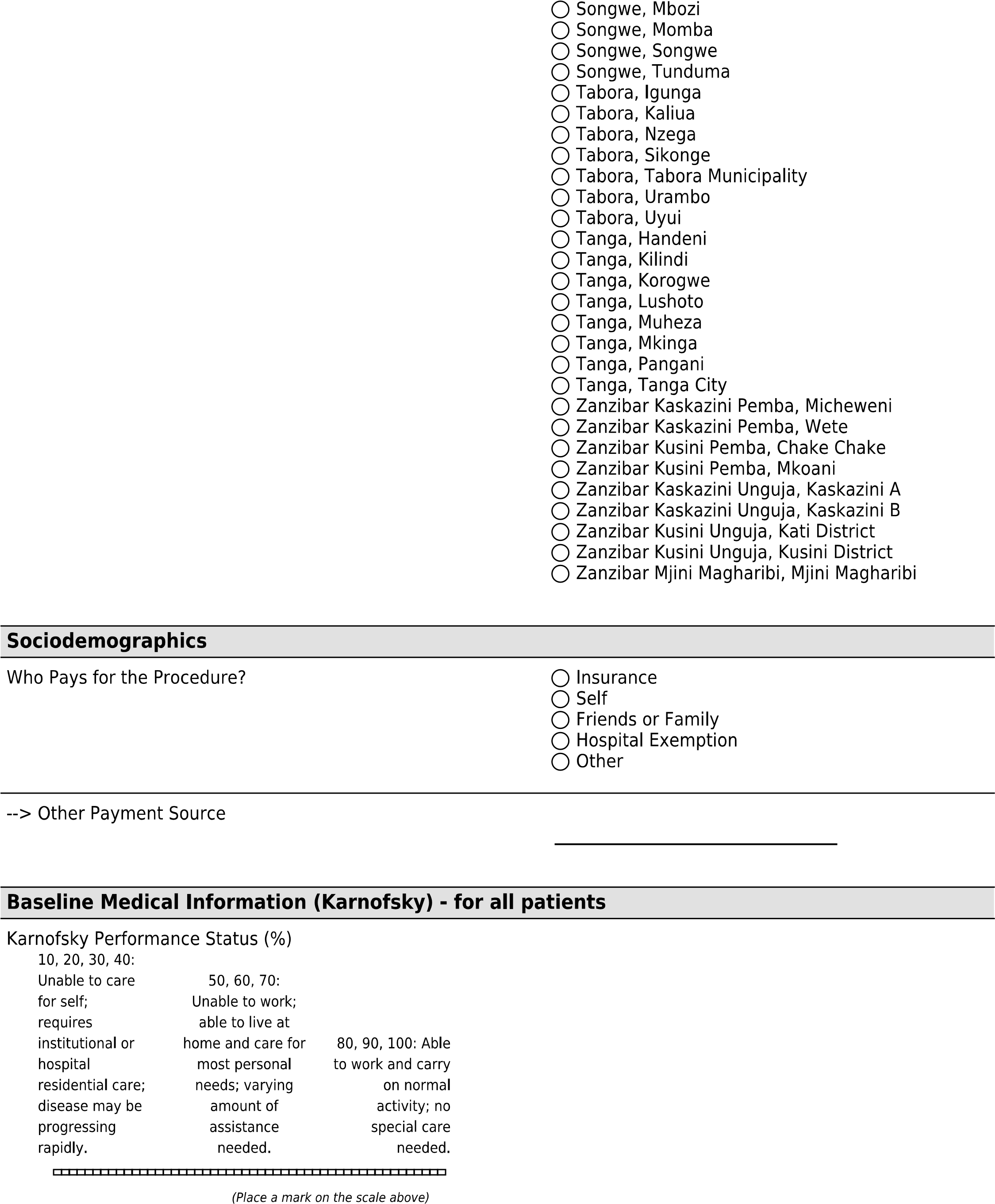


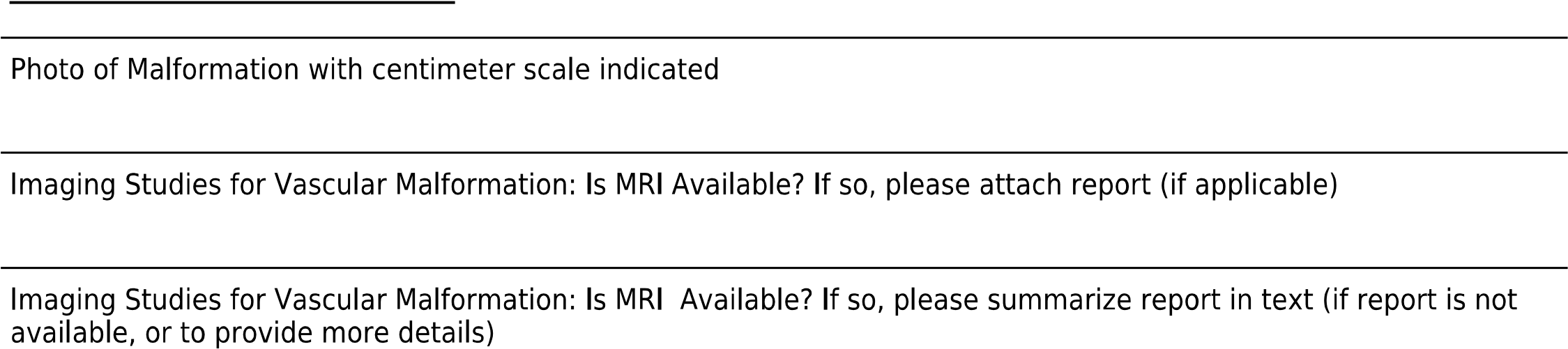


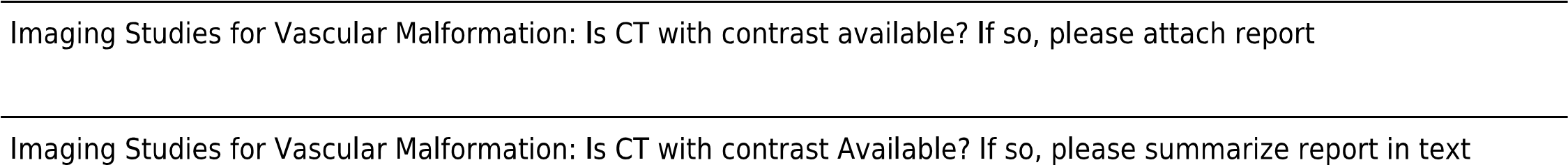


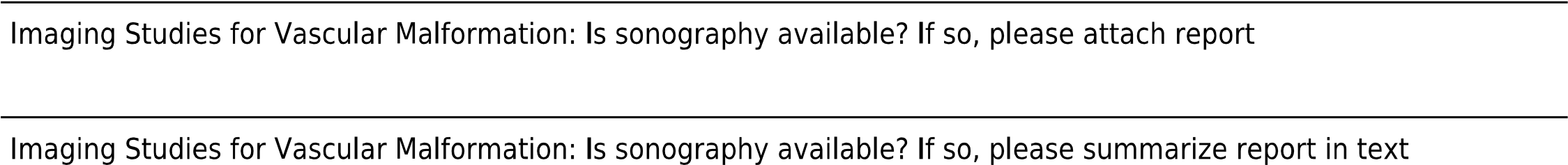


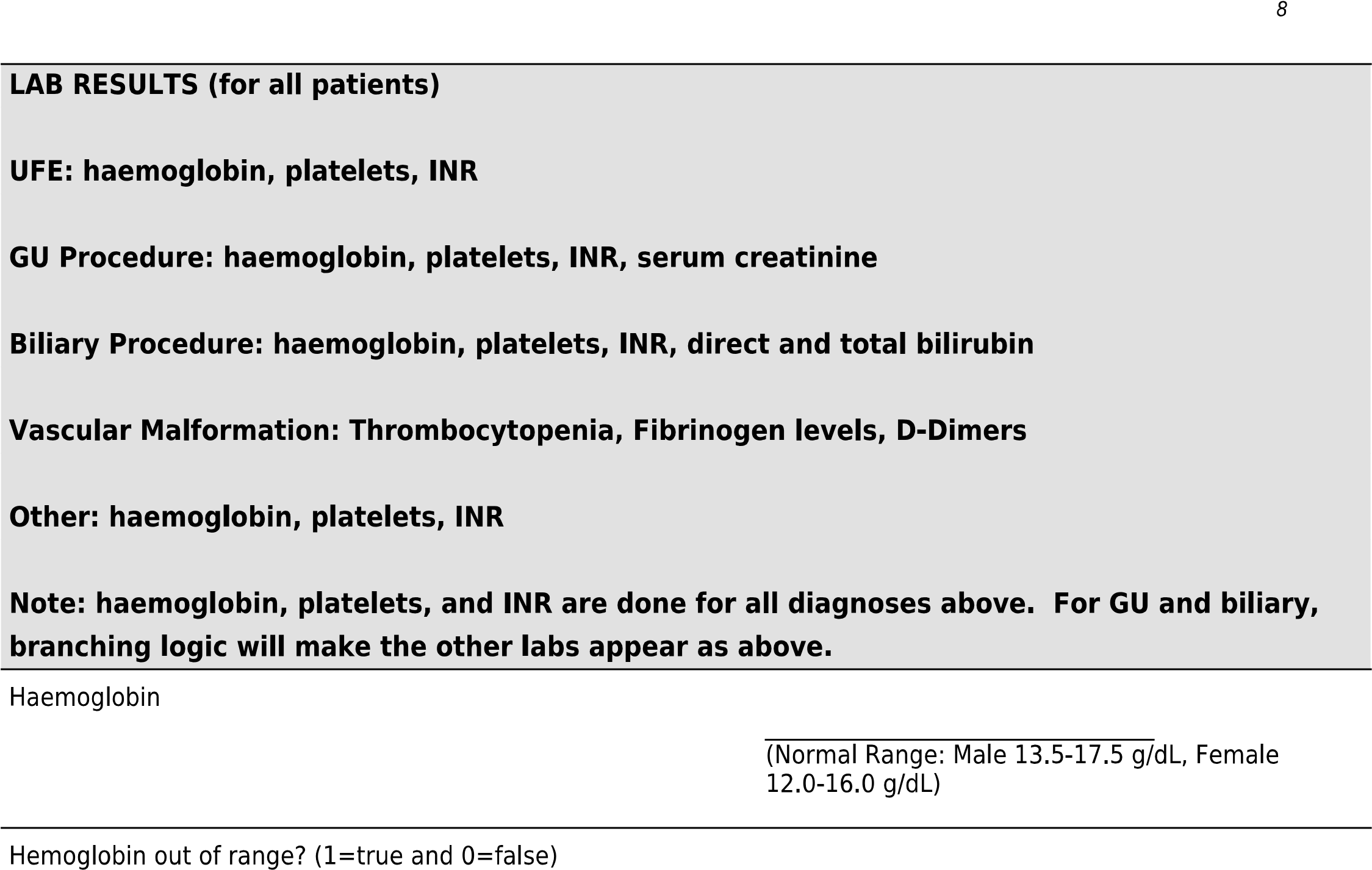


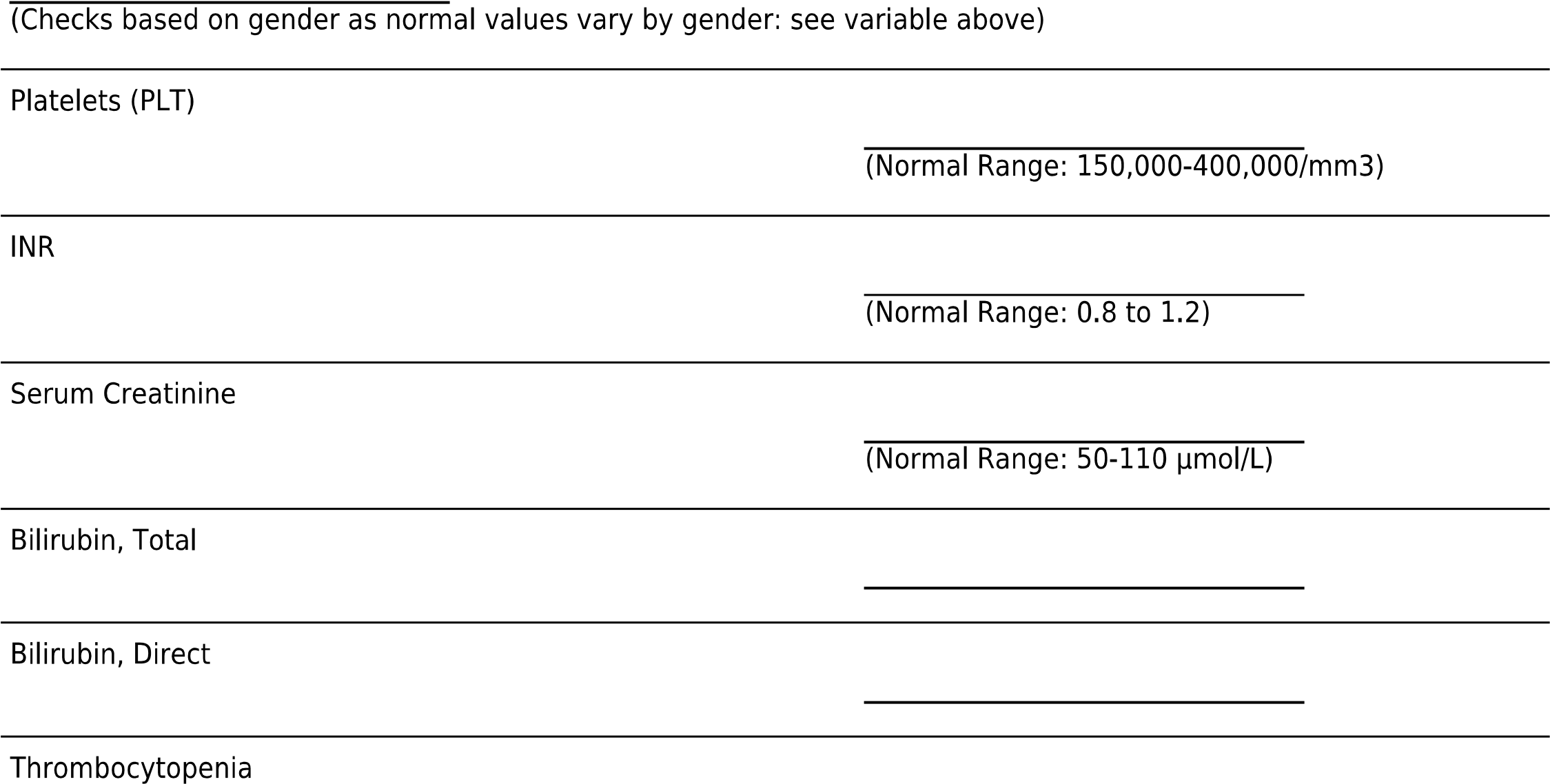


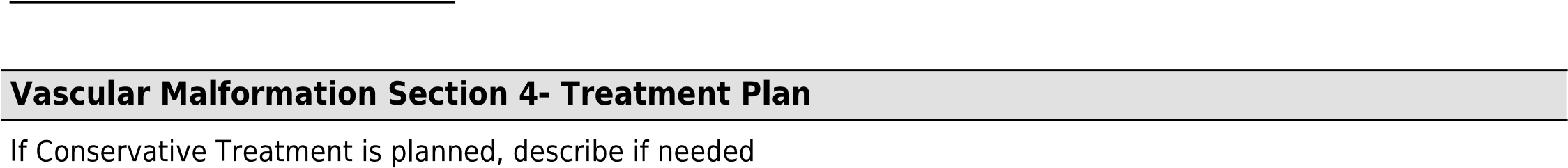


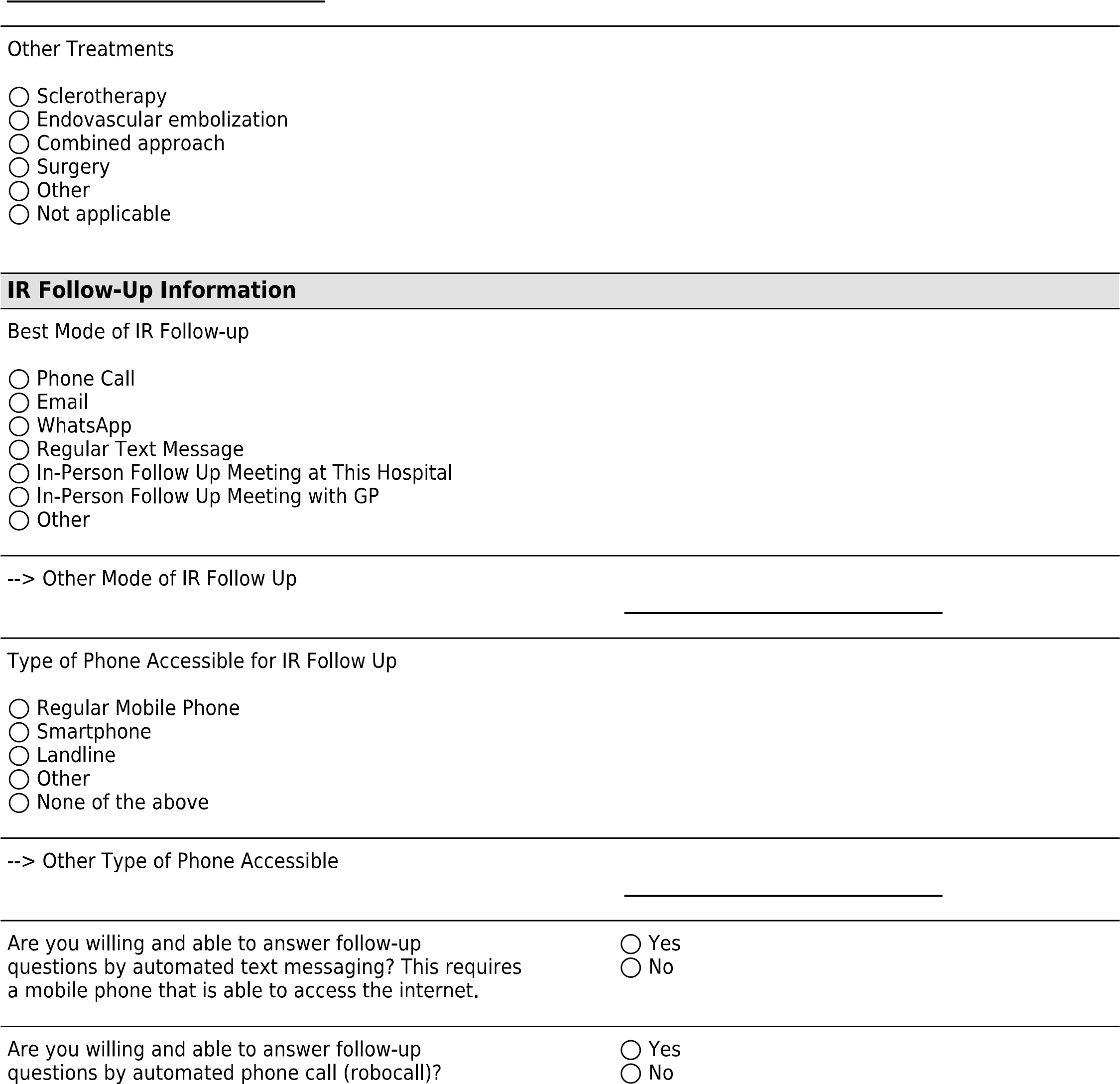


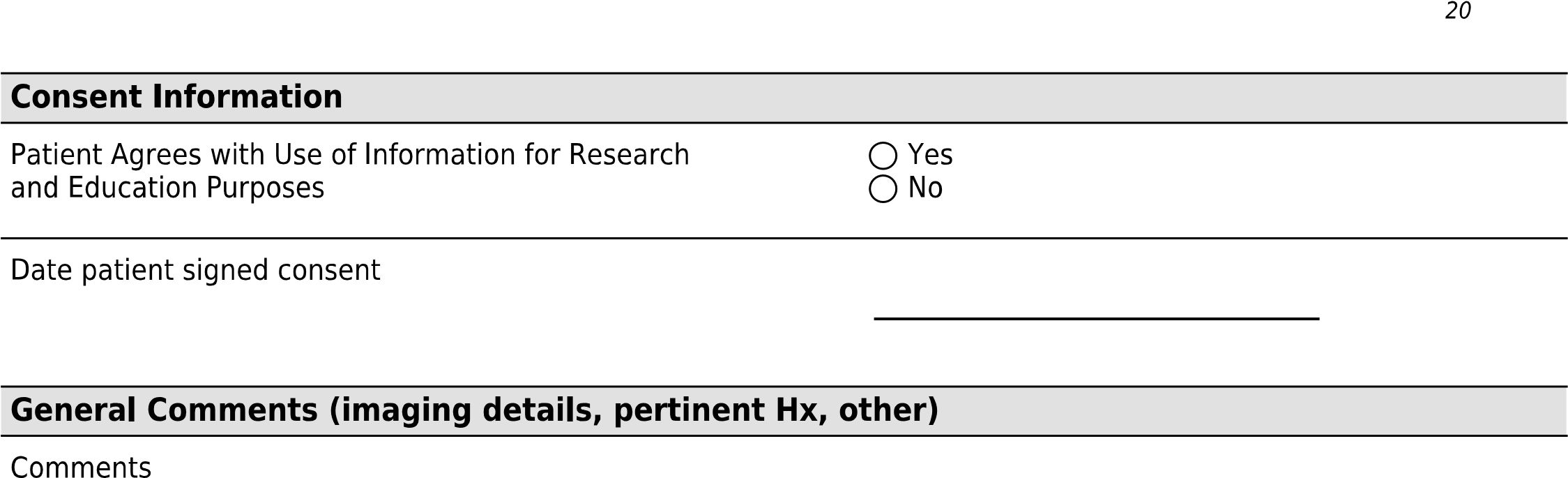

Supplement: Supplementary file 1 [file Table_1.DOCX]
